# A GIMEMA survey on therapeutic use and response rates of FLT3 inhibitors in acute myeloid leukemia: Insights from Italian real‐world practice

**DOI:** 10.1002/jha2.1045

**Published:** 2024-11-22

**Authors:** Monica Messina, Alfonso Piciocchi, Leonardo M. Siena, Stefano Soddu, Francesco Buccisano, Cristina Mecucci, Giovanni Martinelli, Antonio Curti, Roberto Cairoli, Paola Fazi, Marco Vignetti, Maria Teresa Voso, Adriano Venditti, Anna Candoni

**Affiliations:** ^1^ Fondazione GIMEMA Rome Italy; ^2^ Department of Public Health and Infectious Diseases Sapienza University of Rome Rome Italy; ^3^ Hematology Unit, Department of Biomedicine and Prevention University Tor Vergata Rome Italy; ^4^ Hematology and Bone Marrow Transplantation Unit, Department of Medicine and Surgery University of Perugia Perugia Italy; ^5^ IRCCS Azienda Ospedaliero‐Universitaria di Bologna Istituto di Ematologia “L. & A. Seràgnoli” Bologna Italy; ^6^ Department of Hematology ASST Grande Ospedale Metropolitano Niguarda Milan Italy; ^7^ School of Medicine and Surgery Università degli studi di Milano Bicocca Milan Italy; ^8^ Section of Haematology, Department of Medical and Surgical Sciences University of Modena and Reggio Emilia Modena Italy

**Keywords:** acute myeloid leukemia, FLT3 inhibitors, gilteritinib, midostaurin, real‐world

## Abstract

Given the limited data on the real‐life therapeutic use of feline McDonough sarcoma (FMS)‐like tyrosine kinase 3 (FLT3) inhibitors in Italy, we surveyed investigators at 59 Italian hematology centers to gain insight into the proportion of acute myeloid leukemia (AML) patients receiving FLT3 inhibitors and we collected data on the efficacy and safety of these agents. The survey results showed that in the real‐life setting the response rate of the 3/7 + midostaurin regimen in newly diagnosed FLT3‐mutated AML and of gilteritinib in the relapsed/refractory AML were comparable to that reported in the registrative clinical trials. The 3/7 + midostaurin treatment resulted in a 63% of complete remission (CR) rates and gilteritinib in a 44% of CR rates. The discontinuation rate of gilteritinib for intolerance or toxicity was low (accounting for 4% of treated cases).

## 1 INTRODUCTION

FMS‐like tyrosine kinase 3 (FLT3) is one of the most commonly mutated genes in acute myeloid leukemia (AML) [1]. The FLT3 inhibitors midostaurin and gilteritinib have received EMA and Food and Drug Administration (FDA) approval for treatment‐naive FLT3‐mutated AML and relapsed/refractory (R/R) FLT3‐mutated AML, respectively.

The randomized phase III RATIFY trial demonstrated the efficacy of midostaurin in combination with the standard backbone regimen of cytarabine and daunorubicin (3/7) induction and high‐dose cytarabine consolidation in patients younger than 60 years with previously untreated FLT3 (internal tandem duplication (ITD) and/or tyrosine kinase domain (TKD) mutated) AML [2].

In R/R FLT3‐positive AML, the phase III ADMIRAL trial showed significantly longer survival and higher response rates in the gilteritinib arm compared to salvage chemotherapy arm [3]. Consequently, gilteritinib has become the new standard of care for R/R FLT3‐mutated AML.

To date, there is very limited data on the real‐life use of FLT3 inhibitors. Therefore, the aim of our survey was to evaluate the management of FLT3‐mutated AML patients not included in clinical trials in Italy and, in addition, we aimed to assess during the period 2021–2023: (a) the proportion of patients receiving FLT3 inhibitors, (b) the efficacy (in terms of response rates) of FLT3 inhibitors, (c) the rate of AML patients discontinuing treatment due to intolerance or side effects, (d) the rate of early deaths, and (e) the rate of patients receiving FLT3 inhibitors as a bridge to allogeneic transplantation (hematopoietic stem cell transplantation (HSCT)).

## 2 METHODS

Survey data were collected and managed using the REDCap electronic data capture tools hosted at the GIMEMA (the Italian Adult Haematological Diseases Group) Foundation [4]. The invitation to participate in the survey was sent in September 2023 and the data were exported in November 2023. The questionnaire was completed by clinicians of 59 hematology departments of Italian hospitals, belonging to the GIMEMA network, 47 of them also members of the LabNet AML network (Supporting Information Table 1). The survey included the following topics: treatment strategy in patients with FLT3‐mutated AML, response rate of treatment with FLT3 inhibitors, bridge to transplantation rate, and discontinuation of treatment. The survey covered the period from January 2021—when gilteritinib was approved by the Italian Medicines Agency—to September 2023. This survey did not record individual patient data. Therefore, no survival data are available.

## 3 RESULTS

In clinical practice, FLT3 mutation screening is routinely conducted at diagnosis in 93% of the participating centers (55/59). Additionally, 95% of centers (56/59) usually investigate FLT3 mutations in all cases of R/R AML. Sanger and next‐generation sequencing (NGS) are equally used by the Italian hematology centers. During the period 2021–2023, 4773 AML cases were diagnosed in adult patients at the surveyed centers, of which 911 (19%) were FLT3‐mutated. In addition, 389 cases of R/R FLT3‐mutated AML were identified during the observation period.

**FIGURE 1:**
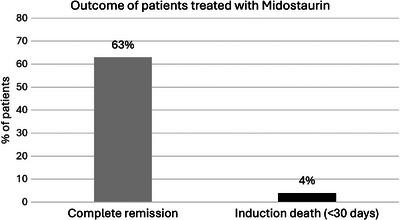
Complete remission rate and induction deaths of newly diagnosed FMS‐like tyrosine kinase 3 (FLT3)‐mutated acute myeloid leukemia (AML) patients treated with midostaurin.

At diagnosis, a significant proportion of FLT3‐mutated AML patients, 62% (570 of 911), were treated with the 3/7 + midostaurin induction chemotherapy regimen. Of those treated, 36% (207) were over 60 years of age. Complete cytological remission (CR) was achieved in 361 patients (63%) and only 21 patients (4%) died within 30 days of induction (Figure 1). Furthermore, 279 (49%) patients underwent subsequent HSCT.

The most common approaches used by the survey participants as an alternative to 3/7 + midostaurin induction were nonintensive chemotherapy with demethylating agents (HMAs) ± venetoclax, reported by 39 centers, or other intensive chemotherapy regimens (without FLT3 inhibitors) such as fludarabine, Ara‐C, idarubicin (FLAI) regimen or CPX‐351, reported by 18 centers.

During the survey period, 389 cases of FLT3‐mutated R/R AML were recorded and 326 patients (84%) started therapy with gilteritinib. Alternatives to salvage therapy with gilteritinib included nonintensive chemotherapy with hypomethylating agent (HMAs) ± venetoclax, reported by 13 centers, while 12 centers reported the use of other intensive chemotherapy regimens (without FLT3 inhibitors) such as FLAI/FLAG (fludarabine Ara‐C, G‐CSF) regimen.

**FIGURE 2:**
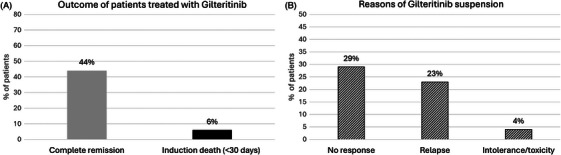
(A) Complete remission rate and induction deaths of relapsed/refractory (R/R) FMS‐like tyrosine kinase 3 (FLT3)‐mutated acute myeloid leukemia (AML) patients treated with gilteritinib. (B) Reasons of gilteritinib suspension.

Among patients treated with gilteritinib, 58% (188/326) were over 65 years old (27% >70 years old). Of the 326 patients, 226 (69%) received the drug for relapse and 100 (31%) for refractory disease. Eighteen percent of patients treated with gilteritinib received the drug for R/R AML following HSCT. Fifty‐four percent (177/326) of patients treated with gilteritinib had been previously treated with midostaurin. CR was achieved in 145 (44%) patients and only 21 patients (6%) died during reinduction (Figure 2A). Sixty (18%) patients received gilteritinib as a bridge to HSCT and this strategy was successful in 51 (85%) patients who proceeded to transplantation in a condition of CR. Overall, approximately half of the treated patients (170, 52%) discontinued treatment with gilteritinib due to relapse or nonresponse during the analyzed period (Figure 2B). Dose reductions due to intolerance/toxicity were reported in 60 patients (18%), while permanent discontinuation for these reasons occurred in only 14 patients (4%; Figure 2B). Discontinuations occurred predominantly within the first 6 months of treatment and, to a lesser extent, between months 6 and 12. In particular, 62% discontinued treatment within 6 months, 12% received gilteritinib for 6–12 months and only 6% for more than 12 months.

## 4 DISCUSSION

This survey shows the approach used to treat FLT3‐mutated AML in Italy in real‐life in the 2021–2023 timeframe, both at diagnosis and in the R/R setting [1]. According to the survey results, the real‐world CR rate with 3/7 + midostaurin administered to patients at diagnosis was 63%, which is comparable to the positive results of the RATIFY trial—where 58.9% of patients achieved CR—and in line with other available literature data [2, 5].

Alternatives to the midostaurin regimen, such as nonintensive chemotherapy, are likely to be used in the case of unfit patients who are ineligible due to comorbidities or advanced age. For the treatment of adult patients with R/R FLT3‐positive AML, the Italian Medicines Agency (AIFA) approved gilteritinib based on the results of the ADMIRAL trial [3]. In the real‐life, salvage therapy with gilteritinib resulted in a 44% CR rate, consistent with the results of the ADMIRAL trial, where the composite CR rate was 54.3% [3, 6]. More than half of those treated with gilteritinib had previously been treated with midostaurin, confirming the efficacy of the treatment in this subset of patients, as observed in clinical practice by Dumas et al. [7]. The present survey shows that gilteritinib was chosen as elective salvage therapy in R/R FLT3‐mutated AML patients of all ages, including those who were ineligible for HSCT or who had already undergone HSCT. In cases where the drug was given as a bridge to transplantation, 85% of patients successfully underwent HSCT procedure. Gilteritinib treatment also showed good safety results with a low rate of both early deaths (6%)—superimposable to those documented in the ADMIRAL trial—and permanent discontinuations due to intolerance/toxicity (4%).

Although this study provides useful information on the real‐world efficacy of FLT3i in a large cohort of patients, it has several limitations due to the nature of the data. In fact, data from this survey are self‐reported by clinicians and patients’ data are aggregated. This hinders the ability to assess survival outcomes, perform subgroup analyses, and make prognostic evaluations.

In conclusion, the results of this survey show how, in a national context, the response rates and safety of the FLT3‐mutated AML treatments are confirmed in a real‐life practice setting. However, in order to provide a more comprehensive and in‐depth evaluation (including survival analysis and prognostic parameters), these survey results should be confirmed in a clinical study.

## AUTHOR CONTRIBUTIONS

Monica Messina analyzed data and wrote the manuscript; Alfonso Piciocchi formally analyzed data and revised the manuscript; Leonardo M. Siena wrote the manuscript; Stefano Soddu formally analyzed data; F. Buccisano, Cristina Mecucci, Giovanni Martinelli, Antonio Curti, Roberto Cairoli, Maria Teresa Voso, and Adriano Venditti provided data and reviewed the manuscript; Paola Fazi and Marco Vignetti administered the project, Anna Candoni designed the project, revised, and edited the manuscript.

## CONFLICT OF INTEREST STATEMENT

Anna Candoni reports honoraria (consultancy, speaker, and advisory role) and/or travel support from AbbVie, Astellas, Janssen, Jazz, Celgene, Gilead, Pfizer, Incyte, and Amgen, outside the submitted work. Francesco Buccisano reports honoraria (speaker and advisory role) from Jazz, Bristol Meyrs Squibb, Janssen‐Cilag, Servier, Astellas, Novartis, Abbvie, and Laboratories Delbert. Antonio Curti reports honoraria (research support, advisory role, and meeting) from Abbvie, Pfizer, Menarini Stem line, Jazz Pharma, Servier outside the submitted work. Roberto Cairoli reports honoraria (speaker and advisory board) from Abbvie, Novartis, Celgene, DaiiChi Sankyo outside the submitted work. Marco Vignetti reports honoraria (advisory role and speaker) from Jazz Healthcare, Millennium Pharmaceuticals, Amgen, Abbvie, and AstraZeneca, Novartis outside the submitted work.

## FUNDING INFORMATION

The authors received no specific funding for this work.

## ETHICS STATEMENT

The authors have confirmed ethical approval statement is not needed for this submission.

## PATIENT CONSENT STATEMENT

The authors have confirmed patient consent statement is not needed for this submission.

## CLINICAL TRIAL REGISTRATION

The authors have confirmed clinical trial registration is not needed for this submission.

## Supporting information



Supporting Information

## Data Availability

Data are available upon request.
