# Supplementary material for: A GIMEMA survey on therapeutic use and response rates of FLT3 inhibitors in acute myeloid leukemia: Insights from Italian real‐world practice
Source: EJHaem. 2024 Nov 22;5(6):1274–7. doi: 10.1002/jha2.1045 (PMC11647686; doi:10.1002/jha2.1045)
Supplement: Supplementary file 1 — Supporting Information [file JHA2-5-1274-s001.docx]

**Supplemental material.**

**Supplemental table 1.** List of the GIMEMA centers and Investigators who completed the survey.

| 165 - AVERSA, Annunziata Mario | 106 - IFO - ROMA, Mengarelli Andrea |
| --- | --- |
| 056 - ASL TARANTO, Aprile Lara | 193 - MILANO - OSPEDALE SAN CARLO, Montefusco Vittorio |
| 037 -MOLINETTE - TORINO - SC EMATOLOGIA 2, Audisio Ernesta | 019 - VILLA SOFIA CERVELLO - PALERMO, Mulè¨ Antonino |
| 086 - MESTRE - UO EMATOLOGIA, Bassan Renato | 099 - CASA DI CURA LA MADDALENA S.P.A. - PALERMO, Musso Maurizio |
| 041 - AS DELLALTO ADIGE, OSPEDALE CENTRALE DI BOLZANO, Billio Atto | 107 - ROVIGO, Paolini Rossella |
| 067 - SIENA, Bocchia Monica | 064 - BRINDISI, Pastore Domenico |
| 012 -NIGUARDA - MILANO, Cairoli Roberto | 129 - TRICASE, Pavone Vincenzo |
| 085 - MODENA, Candoni Anna | 121 - MATERA, Pizzuti Michele |
| 159 - LIVORNO, Capochiani Enrico | 103 - CAMPUS BIO MEDICO - ROMA, Rigacci Luigi |
| 088 - ROMA - S.Filippo Neri, Caravita Di Toritto Tommaso | 059 - ASL Napoli 1, Rocino Angiolina |
| 027 - GEMELLI IRCCS - ROMA, Chiusolo Patrizia | 007 - CAGLIARI, Romani Claudio |
| 170 - MAURIZIANO UMBERTO I - TORINO, Cilloni Daniela | 087 - RAVENNA, Rondoni Michela |
| 153 - ARNAS GARIBALDI - CATANIA, Consoli Ugo | 031 - SAN GIOVANNI ROTONDO, Rossi Giovanni |
| 108 - ISTITUTO NAZIONALE TUMORI - MILANO, Corradini Paolo | 172 - CATANZARO, Rossi Marco |
| 006 - BOLOGNA - POLICLINICO S. ORSOLA-MALPIGHI, Curti Antonio | 038 - AOU DI PARMA, Roti Giovanni |
| 044 - AOU SAN LUIGI GONZAGA - ORBASSANO, De Gobbi Marco | 134 - ASST DEGLI SPEDALI CIVILI DI BRESCIA - CENTRO TRAPIANTI, Russo Domenico |
| 008 - PO OSPEDALIERO G. RODOLICO - CATANIA, Di Raimondo Francesco | 024 - ASL PESCARA, Salutari Prassede |
| 133 - LECCE, OSPEDALE V. FAZZI, Di Renzo Nicola | 010 - AOU CAREGGI - FIRENZE, Scappini Barbara |
| 014 - CARDARELLI - NAPOLI, Ferrara Felicetto | 163 - SALERNO, Selleri Carmine |
| 174 - ASST DEI SETTE LAGHI, VARESE, Ferrario Andrea | 120 - AOU SANT`ANDREA - ROMA, Tafuri Agostino |
| 032 - SASSARI, Fozza Claudio | 137 - BARLETTA, Tarantini Giuseppe |
| 045 - FONDAZIONE IRCCS CA GRANDA - MILANO, Fracchiolla Nicola | 034 - UDINE, Tiribelli Mario |
| 096 - PADOVA, Gurrieri Carmela | 146 - BUSTO ARSIZIO, Todisco Elisabetta |
| 043 -VERONA, Krampera Mauro | 055 - ASST DEGLI SPEDALI CIVILI DI BRESCIA - UO EMATOLOGIA, Tucci Alessandra |
| 115 -NOVARA, Lunghi Monia | 142 - PIACENZA, Vallisa Daniele |
| 147 - ASTI, Marchetti Monia | 301 - TOR VERGATA - ROMA, Venditti Adriano |
| 022 - PERUGIA, Martelli Maria Paola | 181 - CANDIOLO, Vitolo Umberto |
| 028 - ROMA SAPIENZA, Martelli Maurizio | 128 - TRIESTE, Zaja Francesco |
| 026 - REGGIO CALABRIA, Martino Bruno Marino | 058 - ALESSANDRIA, Zallio Francesco |
| 048 - CUNEO, Mattei Daniele |  |
